# 3,3-dimethyl-1-butanol and its metabolite 3,3-dimethylbutyrate ameliorate collagen-induced arthritis independent of choline trimethylamine lyase activity

**DOI:** 10.21203/rs.3.rs-3297018/v1

**Published:** 2023-09-05

**Authors:** Sabrina Fechtner, Brendan E. Allen, Meagan E. Chriswell, Widian K. Jubair, Charles E. Robertson, Jennifer N. Kofonow, Daniel N. Frank, V. Michael Holers, Kristine A. Kuhn

**Affiliations:** University of Colorado Anschutz Medical Campus; University of Colorado Anschutz Medical Campus; University of Colorado Anschutz Medical Campus; University of Colorado Anschutz Medical Campus; University of Colorado Anschutz Medical Campus; University of Colorado Anschutz Medical Campus; University of Colorado Anschutz Medical Campus; University of Colorado Anschutz Medical Campus; University of Colorado Anschutz Medical Campus

## Abstract

Previous studies have identified significant alterations in intestinal carnitine metabolism in mice with collagen-induced arthritis (CIA), potentially linking bacterial dysbiosis with autoimmunity. Bacterial trimethylamine (TMA) lyases metabolize dietary carnitine to TMA, which is oxidized in the liver to trimethylamine-N-oxide (TMAO). TMAO is associated with inflammatory diseases, such as atherosclerosis, whose immunologic processes mirror that of rheumatoid arthritis (RA). Therefore, we investigated the possibility of ameliorating CIA by inhibiting TMA lyase activity using 3,3-dimethyl-1-butanol (DMB) or fluoromethylcholine (FMC). During CIA, mice were treated with 1% vol/vol DMB, 100mg/kg FMC, or vehicle. DMB-treated mice demonstrated significant (>50%) reduction in arthritis severity compared to FMC and vehicle-treated mice. However, in contrast to FMC, DMB treatment did not reduce cecal TMA nor circulating TMAO concentrations. Using gas chromatography, we confirmed the effect of DMB is independent of TMA lyase inhibition. Further, we identified a novel host-derived metabolite of DMB, 3,3-dimethyl-1-butyric acid (DMBut), which also significantly reduced disease and proinflammatory cytokines in CIA mice. Altogether, our study suggests that DMB the immunomodulatory activity of DMB and/or its metabolites are protective in CIA. Elucidating its target and mechanism of action may provide new directions for RA therapeutic development.

## INTRODUCTION

Rheumatoid arthritis (RA) is an autoimmune disease characterized by joint inflammation and destruction and exhibits a worldwide prevalence of 0.8-1.0% [1]. In spite of significant advancements for the treatment of RA, current therapies focus on managing symptoms and slowing disease progression [2]. Unfortunately, 43% of patients do not respond to first line therapy with methotrexate and require more aggressive therapy with biologic medications [3]. Overall, only 60–70% of patients with RA sufficiently respond to therapy within the first six months of administration [4]; after about two years ~ 50% discontinue therapy due to loss of efficacy (35%) or safety (20%), among other issues [5]. Thus, significant limitations in efficacy and safety remain, highlighting the need for improved therapeutics.

A novel therapeutic target for the treatment of RA stems from observations that the initial autoimmunity characteristic of RA may originate at mucosal surfaces [6]. Initial hypotheses for the development of this autoimmunity centered on a microbial-triggered process and later a microbial antigen cross-reactive with self. More recently, specific microbiota in the periodontium and intestine have been associated with RA, either by induction of autoantibody generation or by changes in microbial community composition [7, 8]. Further, animal models suggest that the presence of specific microbiota is necessary for the development of autoimmune arthritis. For example, utilizing the collagen-induced arthritis (CIA) model, mice given broad spectrum antibiotics to deplete their gut microbiomes have reduced intestinal IL-17 and IL-22, as well as reduced circulating IL-6, IL-1β and TNF-α [9]; similar dependencies of the gut microbiome on disease development are observed in the K/BxN and SKG models [10, 11]. These findings suggest that the composition of the gut microbiome and its effects can have an immunomodulatory role in RA pathogenesis. However, the mechanisms of how microbial dysbiosis can promote RA development, and how it may be manipulated for therapy, are not understood.

The microbiome can also influence RA through the effect of bacterially-derived metabolites acting as potent immune modulators. Metabolic assessments of plasma from individuals at-risk for or with RA associate alterations in concentrations of numerous carnitines and choline with the future development or presence, respectively, of RA [12, 13]. Some expanded populations of gut bacteria in RA, including *Prevotella* and *Collinsella*, express TMA lyases that are capable of metabolizing dietary choline and carnitine into a cytotoxic molecule trimethylamine (TMA), which is subsequently absorbed into the host and oxidized primarily by hepatic flavin monooxygenase 3 (FMO3) into non-cytotoxic trimethylamine-N-oxide (TMAO) [7, 14, 15]. Although most of the TMAO produced is excreted from the host, increased plasma TMAO is linked to elevated plasma TNF-α and low-grade inflammation in otherwise healthy controls [16]. TMAO is also associated with disease processes in murine models of atherosclerosis, where it is tied to foam cell formation, NLRP3 inflammasome activation, and generation of the proinflammatory cytokines IL-1β, TNF-α, and IL-6 [17–20]. Notably, patients with RA are at an increased risk of premature death due to accelerated atherosclerosis in cardiovascular comorbidities, while NLRP3 inflammasome activation and production of IL-1β, TNF-α, and IL-6 are also key immune mediators of RA [9, 21 –24]. Individuals at-risk for future RA also demonstrate alterations in production of these key cytokines by circulating monocytes [25]. Therefore, bacterial TMAO production may be a potential therapeutic target in RA.

While FMO3 is not a viable drug target due to risk of drug toxicity, decreased TMAO generation has been reported to be achieved through inhibition of bacterial TMA lyase [26]. Previous studies using the apolipoprotein E (ApoE) knockout (*ApoE^−/−^*) model of atherosclerosis demonstrate that inhibition of TMA lyase via oral administration of the choline structural analogs 3,3-dimethyl-1-butanol (DMB) or fluoromethylcholine (FMC) is beneficial, such that mice given these inhibitors have reduced circulating TMAO levels coupled with reductions in endogenous macrophage foam cell and atherosclerotic lesion formations [26, 27]. However, the benefit of inhibiting TMAO formation in patients with RA is currently unclear. In one study, patients with RA exhibited increased concentrations of excreted TMAO compared to healthy controls, but elevated TMAO was associated with a modest reduction in C-reactive protein (CRP) [28]. In the same study, CIA mice fed a 1% choline diet as a source of TMA/TMAO showed amelioration of disease severity compared to mice given normal chow. Critically, TMAO production from the increased choline consumption was not assessed in this study, and choline itself can act in an anti-inflammatory capacity in both RA and CIA through its involvement in the cholinergic anti-inflammatory pathway [29]. Thus, it is not clear if TMAO is protective or harmful in RA, and directly modulating its production from TMA by inhibiting gut bacterial TMA lyase activity is one relevant approach to resolve this contradiction. Therefore, in this study, we utilized the CIA mouse model of autoimmune arthritis to test our central hypothesis that microbial metabolism of carnitine and choline to TMA/TMAO is a moderator in the development of inflammation and arthritis.

## RESULTS

### DMB but not FMC reduces CIA severity independent of TMA-lyase inhibition

**Figure 1:**
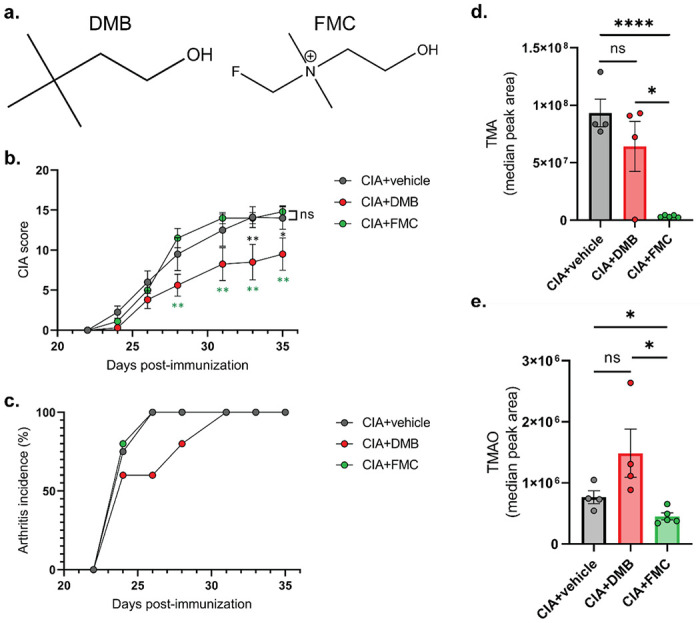
DMB, but not FMC, reduces CIA severity. **(a)** Chemical structures of the TMA lyase inhibitors used in this study. **(b)**CIA was induced in 6-week-old male DBA/1j mice. On day 21 post-initial immunization, mice were treated with either vehicle (CIA+vehicle), 1% (v/v) DMB in drinking water (CIA+DMB), or with 100mg/kg FMC via oral gavage (CIA+FMC). N=4 (CIA), N=4 5 (CIA+DMB), and N=5 (CIA+FMC) per group. Data are reported as mean ± SEM. *, p<0.05; **, p<0.01; ns, non-significant as determined by two-way ANOVA with Bonferroni correction for multiple comparisons. **(c)** Arthritis incidence was calculated by dividing the number of mice showing clinical evidence of arthritis (CIA score ≥ 1) by the total number of mice per group. Data are reported as mean. **(d-e)** Cecal TMA **(d)** and serum TMAO **(e)** concentrations were measured via UHPLC-MS/MS using tissues harvested from mice at day 35 post-initial immunization. N=4 (CIA+vehicle), N=4 (CIA+DMB), and N=5 (CIA+FMC) per group. Data are reported as mean ± SEM. *, p<0.05; ****, p<0.0001; ns, non-significant as determined by unpaired t-test.

Because TMA lyase inhibition using first (DMB) and second-generation (FMC) small molecule inhibitors (Fig. 1a) was effective in ameliorating the inflammatory disease processes underlying the *ApoE^−/−^* mouse model of atherosclerosis [26, 27], we questioned if TMA lyase inhibition using these same inhibitors would also be beneficial in another inflammatory disease model, CIA. Therefore, we induced CIA in male 6-week-old DBA/1j mice. At day 21, mice were treated with vehicle, 1% vol/vol DMB in drinking water, or 100mg/kg FMC via oral gavage as these doses are reported to effectively inhibit TMA generation in mice [26, 27]. Mice were monitored for arthritis until day 35 using the CIA scoring scale described in Methods. Mice given DMB exhibited significantly lower arthritis scores and a delayed incidence of disease compared to untreated and FMC-treated mice (Fig. 1b and 1c). Surprisingly, mice given FMC showed no reduction in disease severity nor a change in arthritis incidence compared to untreated mice. This suggested that DMB reduced joint inflammation through a mechanism divergent from the hypothesized route of TMA lyase inhibition. To determine if DMB and FMC inhibited TMA lyase, ceca and sera were collected from mice at day 35 post-initial immunization and subjected to UPLC-MS/MS analysis for detection of TMA and TMAO. Only FMC treatment resulted in a significant reduction in both cecal TMA and serum TMAO levels compared to vehicle and DMB-treated mice, while mice given DMB displayed no reduction in TMA or TMAO relative to vehicle-treated mice (Fig. 1d and 1e).

**Figure 2:**
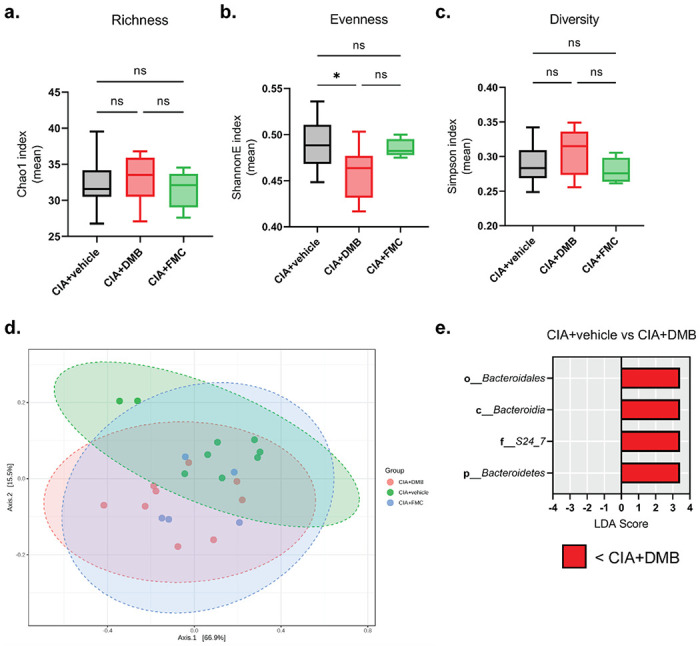
DMB treatment does not confer large-scale alterations in the host gut microbiome. Fecal pellets from DBA/1j mice were harvested at day 35 post-initial immunization and underwent genomic DNA extraction and16S ribosomal RNA gene sequencing to assess microbial diversity. **(a-c)** Alpha diversity analyses of bacterial taxa. N=9 (CIA+vehicle), N=9 (CIA+DMB), N=5 (CIA+FMC) per group and pooled from two independent experiments. Data are reported as box and whisker plots with 5-95% confidence intervals of mean index values. *, p<0.05; ns, non-significant as determined by one-way ANOVA with Tukey’s correction for multiple comparisons. **(d)** PCoA plot showing the beta diversity of microbiota from mice in each treatment group as determined by Bray-Curtis Index distance. N=5-9 per group. Data are shown as individual mice (symbols) with 95% confidence intervals (ellipses). F-value=4.2063; R^2^=0.29608; p-value=0.008 as determined by PERMANOVA between the three treatment groups. **(e)** Linear discriminant effect size analysis of OTU counts from mice treated with vehicle (CIA+vehicle) or DMB (CIA+DMB) using FDR < 0.1, LDA score > 2.0, and FDR-corrected p-value < 0.05. N=9 per group.

Prior studies demonstrated that male *Apoe*^*−/−*^ mice fed a high choline diet developed alterations in cecal bacterial composition, most notably a reduction in the TMA/TMAO-associated Clostridiaceae family, in DMB-treated compared to control-treated mice [26, 27]. Thus, we investigated the hypothesis that a substantial DMB-mediated reorganization of the host gut microbiome led other TMA-producing bacteria, like those from the family Lachnospiraceae that are also expanded during disease development in CIA [9, 30], to compensate for bacteria whose TMA lyases were inhibited by DMB. Fecal pellets from vehicle, DMB, and FMC-treated CIA mice were harvested at day 35 and analyzed by 16S ribosomal RNA gene sequencing for alterations in gut bacterial communities. Measures of alpha diversity indicated that DMB treatment significantly altered microbial evenness (ShannonE), but not richness (Chao1) or diversity (Simpson) compared to vehicle and FMC-treated mice with CIA (Fig. 2a–2c). Further, Bray-Curtis distance analysis of the bacterial communities revealed significant differences in beta diversity among all treatment groups; however, the microbial communities of each treatment group do not substantially separate (Fig. 2d). Non-FDR-corrected Wilcoxon comparisons of bacterial taxa identified a significant expansion of S24-7 and Christenellaceae, and a significant depletion of the genus *Enterorhabdus* in DMB-treated mice compared to vehicle-treated mice (Supplemental Fig. 1a-c). Subsequent linear discriminant effect size analysis with FDR correction, however, identified only a significant expansion of specific taxa driven by S24-7 family bacteria in mice treated with DMB compared to vehicle (Fig. 2e). These alterations in specific taxa largely recapitulate bacterial community changes previously reported during CIA development [9, 30]. Linear discriminant effect size analysis failed to identify significantly altered bacterial taxa between DMB and FMC-treated mice (data not shown); however, Wilcoxon comparisons showed a significant expansion of Ruminococcaceae and significant depletions of Family-XIII-Incertae-Sedis and *Lactobacillus* in DMB-treated mice compared to FMC-treated mice (Supplemental Fig. 1d-f).

While some significant alterations in bacterial communities are present between all treatment groups, overall, the lack of robust changes in DMB-treated mice suggested that it may act independently of bacteria. Thus, we examined the ability of DMB to block the TMA lyase of Proteus *mirabilis* in vitro, which has been previously shown to block conversion of choline to TMA [26, 27]. Whole-cell *P. mirabilis* (ATCC 29906) were cultured with d9-choline and confirmed to produce d9-TMA in a dose-dependent manner (Supplemental Fig. 2a), and subsequently the cells were cultured with a dilution series of DMB (10^− 12^M–10^0^M) for 2 hours. LC-MS/MS analysis of the culture supernatants for production of d9-TMA did not show a dose-dependent reduction in the presence of DMB (Supplemental Fig. 2b). These data suggest that DMB does not universally inhibit TMA lyase and further suggest that its effect in CIA is bacteria independent.

### DMB is absorbed and metabolized in mice

**Figure 3:**
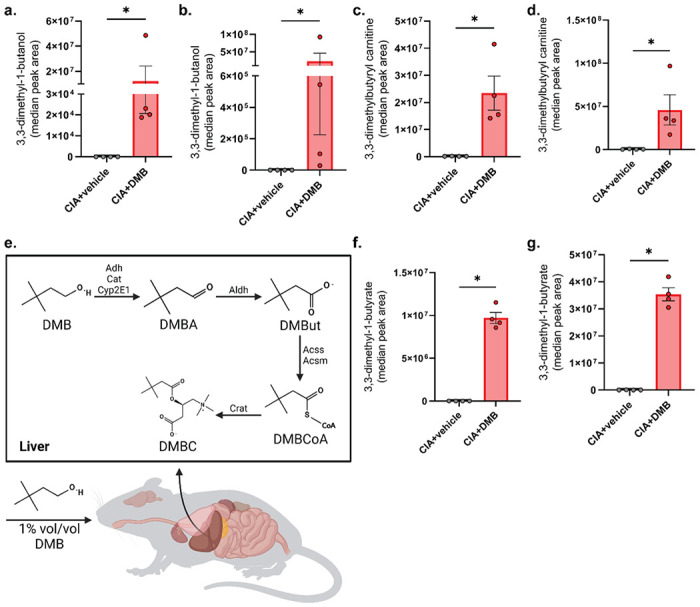
DMB is absorbed into the host and metabolized to its fatty acid and acylcarnitine conjugates. **(a-d)** Serum (**a**, **c**) and liver (**b**, **d**) from male DBA1/j mice was harvested at day 35 post-initial immunization and subjected to GC-MS for detection of DMB (**a-b**) and LC-MS for detection of DMBC (**c-d**). N=4 (CIA+vehicle) and N=4 (CIA+DMB) per group. Data are reported as mean ± SEM. *, p<0.05 as determined by Mann-Whitney non-parametric t-test. **(e)** Hypothesized metabolism of DMB in the liver after it is absorbed into the host. **(f-g)** Serum **(f)** and liver **(g)** was subjected to GC-MS for detection of DMBut. N=4 per group. Data are reported as mean ± SEM. *, p<0.05 as determined by Mann-Whitney non-parametric t-test.

Next, we examined the host metabolism of DMB. GC/MS analysis was used to examine the presence of DMB through identification of three ion peaks corresponding to DMB (Supplemental Fig. 3a). Indeed, mice given DMB had detectable and significantly greater concentrations of DMB in their serum compared to vehicle-treated mice, suggesting absorption into the host (Fig. 3a). Since DMB was administered orally, we hypothesized that DMB is subject to first-pass metabolism in the liver. DMB was detected at significantly higher concentrations in the livers of DMB-treated mice compared to untreated mice (Fig. 3b). Further, previous studies have shown that DMB is a substrate for alcohol dehydrogenase 1 (ADH1) [26]; however, the aldehyde product of DMB generated by ADH1 was undetectable by LC-MS in the livers of DMB-treated mice (data not shown). Instead, the downstream acylcarnitine metabolite, 3,3-dimethylbutyrylcarnitine (DMBC), was detected by LC-MS in the serum and liver (Fig. 3c and 3d), providing evidence for rapid metabolism of the DMB aldehyde to its carboxylic acid conjugate within the host.

In order to further define the metabolism of DMB by the murine host, we mapped the metabolic pathway from DMB to DMBC (Fig. 3e). In the pathway, we predicted the generation of the intermediate metabolite, 3-3-dimethyl-1-butyrate (DMBut). Therefore, we assayed the serum and liver of untreated and DMB-treated mice for the presence of DMBut, which was only present in the serum and liver of CIA mice given DMB but not vehicle-treated mice (Figs. 3f and 3g). The identification of two DMB metabolites within this pathway suggests that DMB is taken up into host tissues where it may be active in disease-modulating processes.

### DMB and DMBut administration reduces inflammatory features of CIA

**Figure 4:**
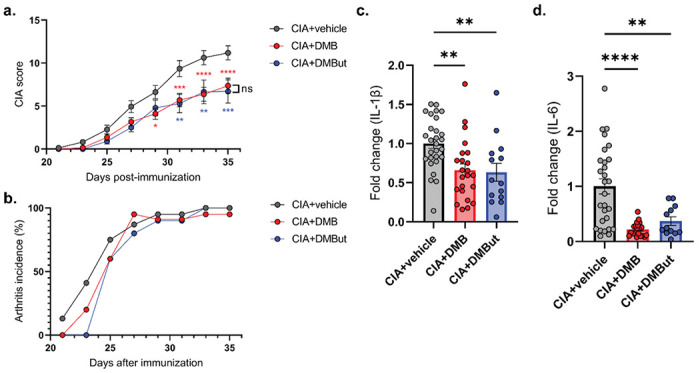
DMB and its metabolite DMBut reduce arthritis severity and proinflammatory cytokines in CIA. **(a)** CIA was induced in 6-week-old male DBA/1j mice. On day 21 post-initial immunization, mice were left untreated (CIA), treated with 1% (v/v) DMB in drinking water (CIA+DMB), or treated with 1% (v/v) DMBut in drinking water (CIA+DMBut). N=24 (CIA), N=23-25 (CIA+DMB), and N=10 (CIA+DMBut) per group pooled from 5 individual experiments. Data are reported as mean ± SEM. *, p<0.05; **, p<0.01; ***, p<0.001; ****, p<0.0001; ns, non-significant as determined by two-way ANOVA with Bonferroni correction for multiple comparisons. **(b)** Arthritis incidence was calculated by dividing the number of mice showing clinical evidence of arthritis (CIA score ≥ 1) by the total number of mice per group. N=24 (CIA), N=23-25 (CIA+DMB), and N=10 (CIA+DMBut) per group pooled from 5 individual experiments. Data are reported as mean. **(c-d).** Serum was harvested from male DBA/1j mice at day 35 post-initial immunization and analyzed for proinflammatory cytokines by a 6-plex immunoassay (Mesoscale). N=24-29 (CIA), N=27 (CIA+DMB), and N=14 (CIA+DMBut) per group. Data are reported as fold change normalized to the cytokine concentrations in the CIA group (symbols) and group mean ± SEM. **, p<0.01; ***, p<0.001; ****, p<0.0001 as determined by one-way ANOVA with Dunnett’s correction for multiple comparisons.

Butyrate is a short-chain fatty acid that has been previously described to have anti-inflammatory effects in both RA and CIA, where it suppresses the severity of disease through several mechanisms, including: regulating differentiation of regulatory B (Bregs) and T cells (Tregs); indirectly amplifying aryl-hydrocarbon receptor activation in Bregs; reducing proinflammatory cytokine and anti-CII antibody production; increasing IL-10 production; and inhibiting osteoclastogenesis [31–36]. Therefore, we questioned whether the host-derived DMBut is an active metabolite of DMB responsible for the attenuation of disease in DMB-treated mice. To test this hypothesis, we treated mice with CIA with either vehicle, 1% vol/vol DMB, or 1% vol/vol DMBut in the drinking water beginning on day 21 post-initial immunization. Mice were monitored for arthritis until day 35 using the CIA scoring scale described in Methods. Like mice given DMB, mice treated with DMBut had significantly reduced CIA scores compared to untreated mice (Fig. 4a). However, the incidence of arthritis did not differ between groups over time (Fig. 4b). Overall, these results suggest that both DMB and DMBut modulate the severity of inflammatory responses during CIA.

To investigate how DMB and DMBut administration modulate the inflammatory response in CIA, we harvested serum from untreated, DMB-treated, or DMBut-treated mice 35 days post-initial immunization and analyzed circulating proinflammatory cytokines and collagen-type II (CII)-specific antibodies. The cytokines IFN-γ, IL-17A, IL-1β, IL-23, IL-6, and TNF were chosen for their relevance to the pathogenesis of CIA and RA [37–40]. DMB and DMBut-treated mice both had significantly reduced circulating concentrations of IL-1β and IL-6 compared to untreated controls (Figs. 4c and 4d). Individually, DMBut significantly reduced TNF-α and IL-23 compared to untreated mice (Supplemental Fig. 4a and 4b); whereas, DMB treatment significantly reduced IFN-γ (Supplemental Fig. 4c). Interestingly, neither compound significantly reduced serum IL-17A (Supplemental Fig. 4d). Total anti-CII IgG was not affected by DMB or DMBut treatment, and only treatment with DMB significantly reduced the concentration of pathogenic isotype IgG2b anti-CII antibodies compared to vehicle-treated mice (Supplemental Fig. 4e and 4f). Thus, DMB and DMBut appear to have the most profound effect on the cytokines IL-1β and IL-6.

**Figure 5:**
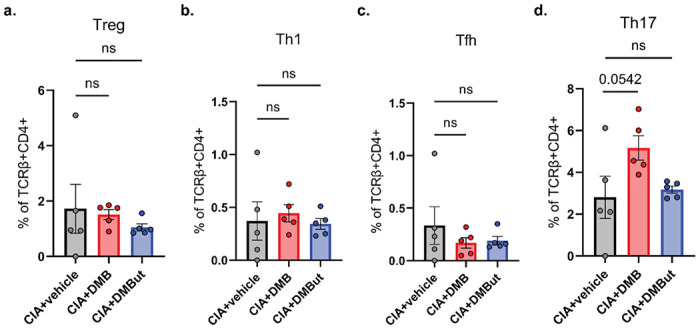
DMB and DMBut do not promote differentiation of regulatory lymphocyte populations in CIA. **(a-d)** Splenocytes were harvested from male DBA/1j mice 35 days post-initial immunization and underwent flow cytometry analysis to assess lymphocyte differentiation in untreated (CIA), DMB-treated (CIA+DMB), and DMBut-treated (CIA+DMBut) mice. N=4-5 (CIA+vehicle), N=5 (CIA+DMB), and N=5 (CIA+DMBut). Data are reported as individual mice (symbols) and the group mean ± SEM. ns, non-significant as determined by one-way ANOVA with Dunnett’s correction for multiple comparisons.

Since butyrate is associated with the expansion of Treg subsets and modulating the balance of helper T cells and regulatory T cells, particularly the Th17/Treg ratio, we next sought to investigate changes in T cell differentiation during CIA in response to DMB and DMBut treatment [32, 33, 36]. Flow cytometric analysis of splenocytes for T lymphocyte populations (Supplemental Fig. 5) showed that mice treated with DMB or DMBut had no changes in the percent of regulatory, Th1, follicular helper, or Th17 T lymphocytes compared to vehicle controls (Figs. 5a–d). Altogether, these data suggest that DMB and DMBut are likely to affect innate responses as indicated by reductions in cytokines IL-1β and IL-6 rather than T cell responses.

### DMB and DMBut reduce macrophage secretion of proinflammatory cytokines

**Figure 6:**
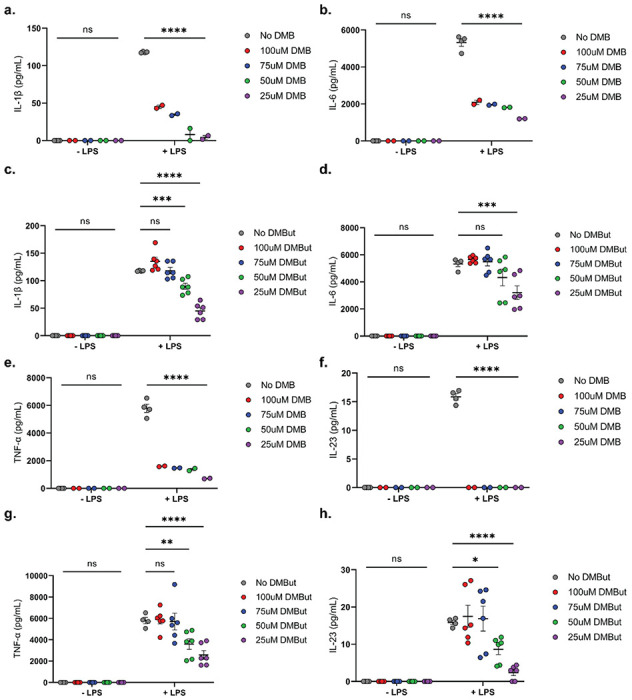
DMB and DMBut reduce production of proinflammatory cytokines from murine BMDMs. **(a-h)** Bone marrow-derived macrophages differentiated from hematopoietic cells isolated from DBA/1j mice were cultured in the presence of 0, 25, 50, 75, or 100μM DMB **(a, b, e, f)** or DMBut **(c, d, g, h)** with or without stimulation by *E. coli* K12 LPS for 24 hours. Supernatants were collected and concentrations of IL-1β **(a, c)**, IL-6 **(b, d)**, TNF-α **(e, g)**, and IL-23 **(f, h).** were analyzed by ELISA. Data are reported as the mean ± SEM of the means two technical replicates pooled from 2 **(a, b, e, f)** or 6 **(c, d, g, h)**independent experiments (symbols). N=4 (0 DMB), N=4 (0 DMB+LPS), N=2 (25μM DMB), N=2 (25μM DMB+LPS), N=2 (50μM DMB), N=2 (50μM DMB+LPS), N=2 (75μM DMB), N=2 (75μM DMB+LPS), N=2 (100μM DMB), N=2 (100μM DMB+LPS), N=4 (0 DMBut), N=4 (0 DMBut+LPS), N=6 (25μM DMBut), N=6 (25μM DMBut+LPS), N=6 (50μM DMBut), N=6 (50μM DMBut+LPS), N=6 (75μM DMBut), N=6 (75μM DMBut+LPS), N=6 (100μM DMBut), and N=6 (100μM DMBut+LPS). *, p<0.05; **, p<0.01; ***, p<0.001; ****, p<0.0001; ns, non-significant as determined by two-way ANOVA with Dunnett’s correction for multiple comparisons.

We next hypothesized that DMB and DMBut interact with monocytes to reduce secretion of IL-1β and IL-6. To investigate this, macrophages derived from the bone marrow of 6 10-week-old DBA/1j mice were cultured with DMB or DMBut in the presence or absence of *E. coli* K12 lipopolysaccharide (LPS). Cells without LPS stimulation showed no IL-1β or IL-6 secretion in response to DMB or DMBut at any concentration in the culture media (Fig. 6a–d). Further, all concentrations of DMB tested reduced secretion of IL-1β and IL-6 from LPS-stimulated macrophages (Fig. 6a and 6b); whereas only those cultured with 50 and 25 μM DMBut reduced secretion of these cytokines (Fig. 6c and 6d). Since mice treated with DMBut, but not DMB, showed reduced serum concentrations of TNF-α and IL-23, we next investigated the effect of DMBut and DMB on secretion of these cytokines from cultured monocytes. Again, cells without LPS stimulation show no TNF-α or IL-23 in response to DMB or DMBut at any concentration (Fig. 6e–h). Surprisingly, all concentrations of DMB tested reduced secretion of TNF-α and IL-23 from LPS-stimulated macrophages (Fig. 6e and 6f); whereas, only those cultured with 50 and 25μM DMBut reduced secretion of these cytokines (Fig. 6g and 6h). On the whole, neither DMB nor DMBut treatment of LPS-stimulated or non-stimulated macrophages significantly reduced viability of these cells at any tested concentration (Supplemental Fig. 6a-b). Notably, both DMB and DMBut exhibited a reverse dose-dependent reduction in cytokine secretion from macrophages such that lower concentrations of each resulted in greater reduction of cytokine.

## DISCUSSION

The initial aim of our study was to elucidate the role of TMA lyase activity in order to propose a novel druggable target for the treatment of RA. To test this, we administered previously characterized TMA lyase inhibitors, DMB and FMC, to mice with CIA and monitored their disease progression. Unexpectedly, blockade of TMA lyase with FMC had no effect on mice with CIA while the significant anti-inflammatory properties of DMB during CIA were found to be independent of TMA lyase activity based on both metabolomic and microbiome analyses. This led us to further investigate the possibility that DMB is absorbed systemically by the murine host. In doing so, we identified novel metabolites, such as DMBut and DMBC, stemming from the host’s metabolism of DMB. Previous studies have shown that butyrate itself is beneficial in RA and CIA [31–35, 41, 42]; therefore, we hypothesized that DMBut is an immunomodulatory metabolite of DMB. Indeed, the administration of DMB or DMBut both ameliorated disease in our CIA mouse model, and our data showing significant reductions in serum IL-1β and IL-6 without significant effects on autoantibody or T lymphocyte subsets suggested that DMB alters innate immune responses.

Our initial finding that DMB did not inhibit TMA lyase has been corroborated by the findings of other studies published since DMB was first characterized as a putative TMA lyase inhibitor. *In vitro* incubation of live bacteria from human fecal samples, recombinant *Desulfovibrio alaskensis* G20 choline trimethylamine-lyase (CutC/D), live *P. mirabilis* ATCC 29906, or *P. mirabilis* ATCC 29906 protein lysate with 10nM–10mM DMB revealed that DMB did not reduce relative CutC activity or production of TMA from choline [43–45]. Moreover, using 1% (v/v) DMB as an inhibitor of *in vivo* TMAO production in numerous disease models has resulted in conflicting efficacy outcomes, such that administration of 1% (v/v) DMB has been shown to either reduce or have no effect on circulating TMAO levels [46–63]. The results of our study in conjunction with the findings of these other studies strongly indicates that the amelioration of disease in these models cannot be attributed solely to the suppression of TMAO generation *in vivo*. Rather, the mechanism of DMB action should be considered as independently of TMA/TMAO production. In addition, directly modulating TMAO production with a specific TMA lyase inhibitor, FMC, does not reduce disease severity in CIA. Altogether, our study strongly suggests that the generation of TMAO from the gut microbiome is not a crucial mediator in the development of CIA or RA.

Next, we sought to characterize the metabolism of DMB in mice. DMB is a substrate of ADH *in vitro* and the acylcarnitine conjugate of DMB, DMBC, has been previously identified in the urine of mice given DMB [26]. Our findings present further insights into the metabolism of DMB *in vivo*. The predicted product of ADH activity on DMB is 3,3-dimethylbutric aldehyde [26]; however, we and others were unable to detect this metabolite in host tissues, presumably due to the rapid metabolism of cytotoxic aldehydes. Instead, we were successful in detecting the downstream acylcarnitine, DMBC, which corroborates previous findings. From these, we predicted the generation of the novel molecules originating from the hepatic metabolism of DMB since DMB is administered orally and would be subject to first-pass metabolism. One of these predicted novel DMB metabolites, the carboxylic acid DMBut, was also detectable only in mice treated with DMB. Notably, DMBut was only present in the serum and liver of CIA mice given DMB, but not in the cecum, suggesting it is not microbially derived and lending evidence to support the absorption and metabolism of DMB within the host.

DMB and DMBut together elicited the most profound immunologic effects on circulating IL-6 and IL-1β. Both IL-6 and IL-1β are key mediators in the pathogenesis of both RA and CIA [64, 65]. Our BMDM experiments show DMB and DMBut inhibit LPS-induced IL-1β, IL-6, TNF-α, and IL-23 release in M1-like differentiated macrophages without substantially affecting viability of these cells. Previous reports have recapitulated these findings *in vivo* in other disease models and mouse strains, demonstrating that treatment with DMB reduces circulating and tissue-specific expression of IL-1β, TNF-α, and IL-6 [53, 56, 59–63, 66–70]. Another study in male C57BL/6J mice fed a high-choline diet suggested that treatment with 1.3% (v/v) DMB reduced IL-1β protein expression in heart tissue through acting on cGAS-STING upstream of NLRP3 expression in macrophages, though this effect was attributed to the presumed inhibition of TMAO production by DMB [71]. In light of our results demonstrating that DMB is present in the host both in its native form and its fatty acid and acylcarnitine conjugates, the effect of DMB and its metabolites on inflammasome activation and IL-1β processing, independent of TMA/TMAO production, must be considered. Furthermore, our data showed DMB is more effective than DMBut in BMDMs in an inverse dose dependent manner, suggesting DMB works independently from DMBut and this action on M1-like differentiated macrophages is sensitive to the concentration of DMB. Similarly, butyrate at high concentrations is shown to be pro-inflammatory during acute colitis in mice by stimulating expression of T-bet and IFN-γ in CD4 + T cells, while at low concentrations and steady state it promotes Treg differentiation [72]. Therefore, future studies should be directed towards characterizing the innate immune response in CIA mice given DMBut.

A significant limitation of our study is that the mechanism of DMB and DMBut remains unclear. We presume that the active metabolite DMBut may work similarly to butyrate. In addition to being a histone deacetylase (HDAC) inhibitor, butyrate is also known to activate the GPCRs GPR43 (FFA2), GPR41 (FFA3), and GPR109A, which are expressed in RA-relevant immune cells and mediate anti-inflammatory responses [73]. Therefore, it is possible that DMBut signals through the same receptors to drive amelioration of arthritis severity in CIA. DMB is also reported to inhibit choline dehydrogenase, causing accumulation of choline in liver and kidney tissue, inhibition of choline phosphorylation, and reduction of available choline to cross into the circulation [74]. The choline transporter CTL1 is highly expressed in macrophage-like and fibroblast-like synoviocytes in the synovia and cartilage of patients with RA, and deficient choline uptake through CTL1 in macrophages reduces IL-1β production via attenuation of mitochondrial ATP synthesis, which drives activation of AMPK-mediated mitophagy and termination of NLRP3 inflammasome activation [75, 76]. Thus, DMB and DMBut have multiple potential mechanistic pathways by which they reduce arthritis severity during CIA.

In summary, we identify the small molecules DMB and DMBut as potent agents to decrease the severity of CIA, and strikingly, provide protection after the initiation of disease. Although not a TMA lyase inhibitor as previously published, DMB and DMBut have multiple potential mechanisms by which they reduce circulating IL-1β and IL-6, possibly through histone deacetylase, GPCR, or NLRP3 pathways in innate immune cells. DMB has been shown to be a viable therapy in the context of atherosclerosis, which is a known co-morbidity of RA, partly through inhibiting endogenous foam cell formation. Though it is unclear if DMB is functioning as an inhibitor of TMA lyases, it may function as an inhibitor of the inflammatory mechanisms contributing to both foam cell formation and autoimmune arthritis. Further studies should focus on the effects of DMB/DMBut administration during CIA and the mechanisms by which they influence the inflammatory pathways underlying both RA and atherosclerosis that are independent of TMA/TMAO production.

## METHODS

### Collagen-induced arthritis.

Male 6-week-old DBA/1j mice were injected intradermally at the base of the tail on days 0 and 21 with 100μl of an emulsion containing 200μg nasal bovine type II collagen (CII, Elastin) in 0.01M glacial acetic acid and an equal volume of complete Freund’s adjuvant (Millipore Sigma). On day 21, mice were either left untreated or treated with 1% (vol/vol) DMB (TCI Chemicals) or 1% (vol/vol) DMBut (Sigma-Aldrich) or no additional additive (vehicle), in drinking water with 100mg/ml grape-flavored sugar-sweetened Kool-Aid (Kraft Foods) added to encourage consumption, or 100mg/kg FMC (Jubilant Biosys Limited) gavaged orally every other day. Mice were monitored for onset of arthritis and severity of disease until 35 days after the initial immunization. Disease for each of the four paws was scored on a scale from 0–4 according to established metrics: 0–no erythema or joint swelling; 1–erythema and one swollen digit; 2–erythema and two swollen digits; 3–erythema and three swollen digits; 4–ankylosis. The score for each paw was summed to generate a total score per mouse. The incidence of arthritis was defined as a nonzero score. Therefore, the rate of incidence, as a percentage, indicates how many mice in the treatment group had a non-zero score on a given day. At euthanasia on day 35, blood was collected by cardiac puncture and the serum stored at −20°C for future analysis. Spleens and inguinal lymph nodes were harvested and processed. Feces were collected at day 35 for 16S ribosomal RNA sequencing analysis. Terminal euthanasia of animals involved intraperitoneal injection of a mixture of ketamine (100mg/kg) and xylazine (10mg/kg), with subsequent cardiac puncture as a secondary method of euthanasia.

### Cytokine quantification.

Serum taken from mice with CIA on day 35 post-immunization was analyzed for cytokine concentrations using a Meso Scale Discovery U-Plex assay platform according to manufacturer instructions. Assay plates were imaged on a MESO QuickPlex SQ 120 at the University of Colorado, Anschutz Medical Campus Human Immune Monitoring Shared Resource (HIMSR).

Overnight cell cultures treated with vehicle, DMB, and DMBut with or without the presence of stimulating agents were pelleted at 1660rpm for 5 minutes to collect cell-free supernatants for quantification of secreted cytokine concentrations via ELISA. Concentrations of secreted murine IL-6, IL-17A, IFN-γ, IL-1β, and TNFα were determined using DuoSet ELISA kits (R&D Systems) according to manufacturer instructions at room temperature. Briefly, assay plates were coated with capture antibody diluted to the appropriate working concentrations in PBS overnight and subsequently blocked with 1% bovine serum albumin (BSA) in PBS for 2 hours. Unknown supernatant samples were either undiluted (IL-1β), diluted 1:1 (IL-17A and IFN-γ), or diluted 1:10 (IL-6 and TNFα) in 1% BSA/PBS. Samples incubated on the assay plate for 2 hours. Assay plates were developed in 100μl TMB Substrate Solution (Thermofisher Scientific) for 20 minutes and subsequently stopped with 50μl of 2N H_2_SO_4_ stop solution. Assay plates were imaged immediately after addition of stop solution on a SpectraMax iD5 plate reader at 450nm with correction at 540nm.

### Anti-collagen type II-IgG antibody quantification.

Serum from day 35 post-immunization was evaluated for anti-collagen type II antibody concentrations via enzyme-linked immunosorbent assay (ELISA). All steps were performed on ice. ELISA-grade CII (Chondrex) was diluted 1:10 in 1x collagen dilution buffer (Chondrex) and incubated on the assay plate at 4°C overnight with gentle rocking while covered with aluminum foil. The assay plate was blocked with 0.5% BSA (Sigma-Aldrich) in PBS for 4 hours at 4°C with gentle rocking. A relative standard was generated using serum from a mouse with robust CIA, not otherwise treated, diluted 1:1,000 in 0.5% BSA/PBS and serially diluted 1:4. Unknown serum samples were diluted 1:10,000 in 0.5% BSA/PBS and incubated on the assay plate overnight at 4°C with gentle rocking while covered in aluminum foil. Goat anti-mouse IgG Fab-HRP IgG1-HRP IgG2a-HRP and IgG2b-HRP antibodies (Southern Biotech) were diluted 1:10,000 in PBS and incubated on the assay plate at room temperature for 2 hours with gentle shaking. The assay was developed with 100μl of 1:1 BD OptEIA TMB reagents (BD Bioscience) at room temperature for 20 minutes, and subsequently stopped with 100μl 2N H_2_SO_4_ stop solution. Assay plates were imaged immediately after addition of stop solution on a SpectraMax iD5 plate reader at 450nm with correction at 570nm.

### Flow Cytometry.

Splenocytes were strained through 70μm cell strainers (Fisher Scientific) and washed with serum-free RPMI 1640. The cell suspensions were pelleted at 4°C and 300xg for 5 minutes and the supernatant was discarded. Lymphocytes were resuspended in 1ml 5% fetal bovine serum (FBS) in PBS. Splenocytes were resuspended in 1ml 1x red blood cell lysis buffer (Invitrogen) and incubated on ice for 5 minutes. Lysis was stopped with 10ml PBS and the cell suspension was pelleted at 4°C and 300xg for 5 minutes. Splenocytes were resuspended in 1ml 5% FBS in PBS. 100μl of each cell suspension was added to a 5ml polystyrene round-bottom tube (Corning) and incubated in 1μl Human TruStain FcX (Biolegend) for 5 minutes at 4°C. 10μl Brilliant Stain Buffer Plus (BD Biosciences) was added to each tube and cells were stained for viability and surface markers as noted in Supplemental Table 1. Stained cells incubated at 4°C for 30 minutes. Cells were washed with 1ml 5% FBS in PBS at 4°C and 300xg for 5 minutes, then fixed and made permeable in 1ml Foxp3/Transcription Factor Staining Buffer (Tonbo Biosciences). Cells incubated at 4°C for 30 minutes. Fixed cells were washed twice with 1ml 1x Flow Cytometry Perm Buffer (Tonbo Biosciences) at 4°C and 300xg for 5 minutes and stained for intracellular markers. Stained cells incubated at 4°C for 45 minutes, then washed with 1ml 1x Flow Cytometry Perm Buffer at 4°C and 300xg for 5 minutes and resuspended in 300μl 5% FBS in PBS for analysis. Analysis of data was performed using FlowJo (version 10.8.1). Supplemental Table 2 lists the definitions of the T lymphocyte populations presented.

### TMA lyase inhibition assay

Proteus mirabilis (ATCC 29906) was cultured in 5ml Difco Nutrient Broth (BD Biosciences) overnight at 37°C and 215 rpm without antibiotic selection. Overnight cultures were sub-cultured at a dilution of 1:20 in fresh nutrient broth and grown overnight at 37°C and 215 rpm to serve as the starting material for downstream assays.

Inhibition of the *P. mirabilis* TMA lyase enzyme complex CutC/CutD by DMB was assessed as previously described with some modifications [26, 27, 77]. Briefly, overnight *P. mirabilis* cultures were pelleted by centrifugation at 3000 rpm for 30 minutes and the broth supernatant was discarded. Cells were resuspended in 10 ml PBS and 400μl of cell suspension was allocated to 13x100mm screw cap culture tubes (Pyrex) with gas-tight 13mm-425 Mininert valve caps (Supelco). To determine functionality of endogenous *P. mirabilis* CutC/CutD, bacteria were incubated at 37°C in the presence of 0μM, 25μM, 50μM, 75μM, and 100μM D9-choline (Cambridge Isotope Laboratories) for 2, 4, 6, and 24 hours. To determine the inhibition of endogenous *P. mirabilis* CutC/CutD by DMB, bacteria were incubated in the presence of 1M, 10mM, 100μM, 1μM, 10nM, 100pM, and 1 pM DMB for 15 minutes, then 100μM D9-choline was added to the reaction vials. Reactions were performed at 37°C for 2 hours. Reactions were quenched with 200μl of cold 1M NaOH and submerged in a liquid nitrogen bath. 2ml hexanes, 1ml butanol, and 200μl 1N NaOH were added to the reaction vials, vortexed for 1 minute, and centrifuged for 15 minutes at 4°C and 2500rpm. The upper phase was transferred to a new 13x100mm screw cap culture tube with PTFE-lined caps and 200μl of 0.2N formic acid was added. Vials were vortexed for 1 minute and centrifuged for 15 minutes at 4°C and 2500rpm. The lower aqueous phase was collected and stored at −80°C until analysis by stable isotope dilution LC-MS/MS.

### Bone marrow-derived macrophage differentiation.

Bone marrow-derived macrophages were isolated from 6-10-week-old DBA/1j mice as previously described [78]. Briefly, bone marrow was flushed and processed to a single cell suspension from the femur and tibia using 1ml cold PBS. The single cell suspension was cultured in 9ml of complete RPMI 1640 supplemented with 10% fetal bovine serum, 2% HEPES, 0.6% penicillin/streptomycin, 0.1% 2-mercaptoethanol, and 20ng/ml recombinant mouse GM-CSF (Peprotech). After 72 hours, the cell culture media was replaced with fresh differentiation media to remove non-adherent cells. After 6 days of culture, adherent cells were washed with cold PBS and resuspended in RPMI 1640 without recombinant mouse GM-CSF. Cells were stimulated with 10μg/μl ultrapure *E. coli* K12 lipopolysaccharide (InvivoGen) overnight.

### Gas Chromatography.

Serum samples were thawed to room temperature. After a brief vortex, 50μL of each serum sample was transferred to a glass vial. 3N HCl (20 μL) was added followed by hexane (50 μL) and vortexed thoroughly. The material was then transferred to respective vial inserts (150 μL). All the samples were then centrifuged at 4°C, 10 min, 3000 rpm. The upper hexane layer was then taken out and transferred into respective new vial inserts and capped immediately for chromatography.

Frozen liver samples were ground in a mortar with pestle in liquid N_2_. The suspension of ground liver in liquid N_2_ was quickly poured into a pre-weighed glass vial, allowing the liquid N_2_ to evaporate prior to capping and weighing the sample. To each sample, hexane was added in a weight (mg) to volume (μL) ratio of 1:2 and stored at −20°C. 3N HCl (20 μL) was added to each hexane-suspension, sonicated for 15 min, and vortexed vigorously for 5 min. The 100 μL top liquid layer was then transferred to respective vial inserts and capped. All the samples were then centrifuged at 4°C for 10 min at 3000 rpm. The upper hexane layer (50 μL) was then transferred into a new vial insert and capped immediately for chromatography.

Hexane extracts (1 μL) were injected into a Trace 1310 GC coupled to a Thermo ISQ-LT MS, at splitless mode. The inlet was held at 250°C. Peak separation was achieved on a 30m DB-WAXUI column (J&W, 0.25 mm ID, 0.25 μm film thickness). Oven temperature was held at 80°C for 2 min, ramped at 20°C/min to 125°C, then ramped at 40°C/min to 175°C and then to 240°C at 20°C/min with a final hold for 20 min. Helium carrier gas flow was held at 1.2 mL/min. Temperatures of transfer line and ion source were both held at 250°C. SIM mode was used to scan ions m/z 57, 69, 87 for DMB and m/z 59, 57, 101 for DMBut with scan time of 0.1 sec/ion under electron impact mode. Peak integration was completed using Chromeleon software (ThermoFisher) (Supplemental Fig. 3b and 3c).

### UHPLC-tandem mass spectrometry.

Frozen *P. mirabilis* broth and mouse serum samples were thawed on ice and extracted with ice cold methanol, acetonitrile, and water (5:3:2, respectively) at a 1:25 ratio. Frozen cecum and liver samples were weighed to the nearest 0.1mg and extracted at 15mg/ml in the same extraction buffer.

Extractions were vortexed for 30 minutes at 4°C and then insoluble materials were pelleted by centrifugation at 18,000xg for 10 minutes at 4°C. Supernatants were analyzed using a Thermo Vanquish UHPLC coupled to a Thermo Q Exactive MS and run in positive and negative ion modes (separate runs). Injection volumes were 20μl for serum and broth extracts and 10μl for tissue extracts. UHPLC phases were water (A) and acetonitrile (B) supplemented with 0.1% formic acid for positive mode runs and 1mM ammonium acetate for negative mode runs. Metabolites were separated on a Kinetex C18 column (2.1 x 150mm, 1.7μm, Phenomenex) equipped with a guard column using a 5-minute gradient method with the following conditions: Flow rate 0.45ml/min; column temperature 45°C; sample compartment temperature 7°C; solvent gradient: 0-0.5 minute 5% B, 0.5–1.1 minute 5–95% B, 1.1–2.75 minute hold at 95% B, 2.75-3 minutes 95 – 5% B, 3–5 minute hold at 5% B. The mass spectrometer was operated in full MS mode at a resolution of 70,000, maximum injection time of 200ms, microscans 2, automatic gain control (AGC) ions, electrospray source voltage 4.0 kV, capillary temperature 320°C, and sheath gas 45, auxiliary gas 25, and sweep gas 0 (all nitrogen). Instrument stability and quality control were assessed using replicate injections of a technical mixture every 15 runs as previously described [79, 80]. Raw data files were converted to mzXML using RawConverter and metabolites were annotated and peaks integrated using Maven [81–83] in conjunction with the Kyoto Encyclopedia of Genes and Genomes (KEGG) database.

For D9-trimethylamine (TMA), TMA, and TMAO measurements, the mass spectrometer was operated as above with a scan range of 50–750 m/z. For 3,3-diemethylbutyrylcarnitine measurements, the mass spectrometer was operated as above with a scan range of 65–900 m/z.

### Microbiome analysis.

Bacterial profiles were determined by broad-range amplification and sequence analysis of 16S rRNA genes following our previously described methods [8, 84, 85]. In brief, amplicons were generated using primers that target approximately 400 base pairs of the V3V4 variable region of the 16S rRNA gene. PCR products were normalized using a SequalPrepTM kit (Invitrogen, Carlsbad, CA), pooled, lyophilized, purified and concentrated using a DNA Clean and Concentrator Kit (Zymo, Irvine, CA). Pooled amplicons were quantified using Qubit Fluorometer 2.0 (Invitrogen, Carlsbad, CA). The pool was diluted to 4nM and denatured with 0.2 N NaOH at room temperature. The denatured DNA was diluted to 15pM and spiked with 25% of the Illumina PhiX control DNA prior to loading the sequencer. Illumina paired-end sequencing was performed on the Miseq platform with versions v2.4 of the Miseq Control Software and of MiSeq Reporter, using a 600-cycle version 3 reagent kit.

Illumina Miseq paired-end reads were aligned to human reference genome hg19 with bowtie2 and matching sequences discarded [86, 87]. As previously described, the remaining non-human paired-end sequences were sorted by sample via barcodes in the paired reads with a python script [88]. Sorted paired end sequence data were deposited in the NCBI Short Read Archive under accession number PRJNA1006768. The sorted paired reads were assembled using phrap [89, 90]. Pairs that did not assemble were discarded. Assembled sequence ends were trimmed over a moving window of 5 nucleotides until average quality met or exceeded 20. Trimmed sequences with more than 1 ambiguity or shorter than 350 nucleotides were discarded. Potential chimeras identified with Uchime (usearch6.0.203_i86linux32) using the Schloss Silva reference sequences were removed from subsequent analyses [91, 92]. Assembled sequences were aligned and classified with SINA (1.3.0-r23838) using the 418,497 bacterial sequences in Silva 115NR99 as reference configured to yield the Silva taxonomy [93, 94]. Operational taxonomic units (OTUs) were produced by clustering sequences with identical taxonomic assignments. This process generated 4136760 sequences for 23 samples (median sample size: 169668 sequences/sample; IQR: 113008 to 253290 sequences/sample). The median Goods coverage score was ≥ 99.97%. The software package Explicet (v2.10.5, www.explicet.org) was used for data organization and alpha-diversity calculations [95].

### Statistics.

Unless specified otherwise, data was analyzed using GraphPad Prism software version 9; specific statistical tests for comparisons are referenced in the figure legends.

### Ethics declarations.

All animal studies and methods were approved by the University of Colorado School of Medicine Institutional Animal Care and Use Committee (protocol #173). All animal studies and methods were performed in accordance with the ethics guidelines and regulations put forth by the University of Colorado School of Medicine Institutional Animal Care and Use Committee. All studies are reported in accordance with the ARRIVE 2.0 guidelines [96, 97].

## Data Availability

16S sequencing data are publicly deposited under BioProject accession: PRJNA1006768 (https://www.ncbi.nlm.nih.gov/bioproject/PRJNA1006768). All other data are available upon request to the corresponding author.
